# Polarization-controlled directional scattering for nanoscopic position sensing

**DOI:** 10.1038/ncomms11286

**Published:** 2016-04-20

**Authors:** Martin Neugebauer, Paweł Woźniak, Ankan Bag, Gerd Leuchs, Peter Banzer

**Affiliations:** 1 Max Planck Institute for the Science of Light, Günther-Scharowsky-St. 1, D-91058 Erlangen, Germany; 2 Institute of Optics, Information and Photonics, Department of Physics, Friedrich-Alexander-University Erlangen-Nuremberg, Staudtstrasse 7/B2, D-91058 Erlangen, Germany; 3 Department of Physics, University of Ottawa, 25 Templeton Street, Ottawa, Ontario K1N 6N5, Canada

## Abstract

Controlling the propagation and coupling of light to sub-wavelength antennas is a crucial prerequisite for many nanoscale optical devices. Recently, the main focus of attention has been directed towards high-refractive-index materials such as silicon as an integral part of the antenna design. This development is motivated by the rich spectral properties of individual high-refractive-index nanoparticles. Here we take advantage of the interference of their magnetic and electric resonances to achieve strong lateral directionality. For controlled excitation of a spherical silicon nanoantenna, we use tightly focused radially polarized light. The resultant directional emission depends on the antenna's position relative to the focus. This approach finds application as a novel position sensing technique, which might be implemented in modern nanometrology and super-resolution microscopy set-ups. We demonstrate in a proof-of-concept experiment that a lateral resolution in the Ångström regime can be achieved.

## Abstract (web summary)

High-refractive-index nanoantennas support magnetic and electric resonances that can be excited with structured light. Here, the authors exploit the interference of such resonances to achieve strong lateral directionality of the emission and utilize this effect for nanoscopic position sensing.

## Graphical abstract

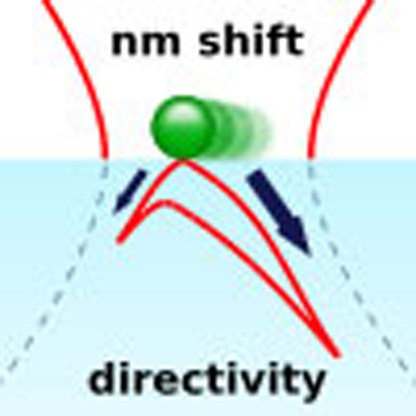

Cylindrical vector beams are well-established tools in modern microscopy, ranging from scanning microscopy, where a reduced focal spot size can be achieved with a radially polarized beam^1^,^2^,^3^,^4^, to more sophisticated techniques such as stimulated-emission depletion^5^,^6^ and multi-photon microscopy^7^. In addition, those polarization-tailored beams have also paved the way towards versatile applications in recent nanophotonic experiments by enabling selective excitation of nanoparticle eigenmodes^8^,^9^ or controllable directional emission and waveguide-coupling of single plasmonic nanoantennas^10^.

In this work, we combine several aspects of both research fields to present a novel approach towards high-precision position sensing, a discipline that is of paramount importance in modern nanometrology^11^,^12^,^13^,^14^,^15^,^16^,^17^,^18^, because of its special role in super-resolution microscopy^19^,^20^,^21^,^22^. Our all-optical technique for localization of a single nanoantenna is thereby based on encoding the position of the antenna in its laterally directional scattering pattern. For that purpose, we take advantage of the resonance properties of a high-refractive-index silicon nanoantenna featuring electric and magnetic resonances^23^,^24^,^25^,^26^.

## Results

### Excitation scheme

**Figure 1:**
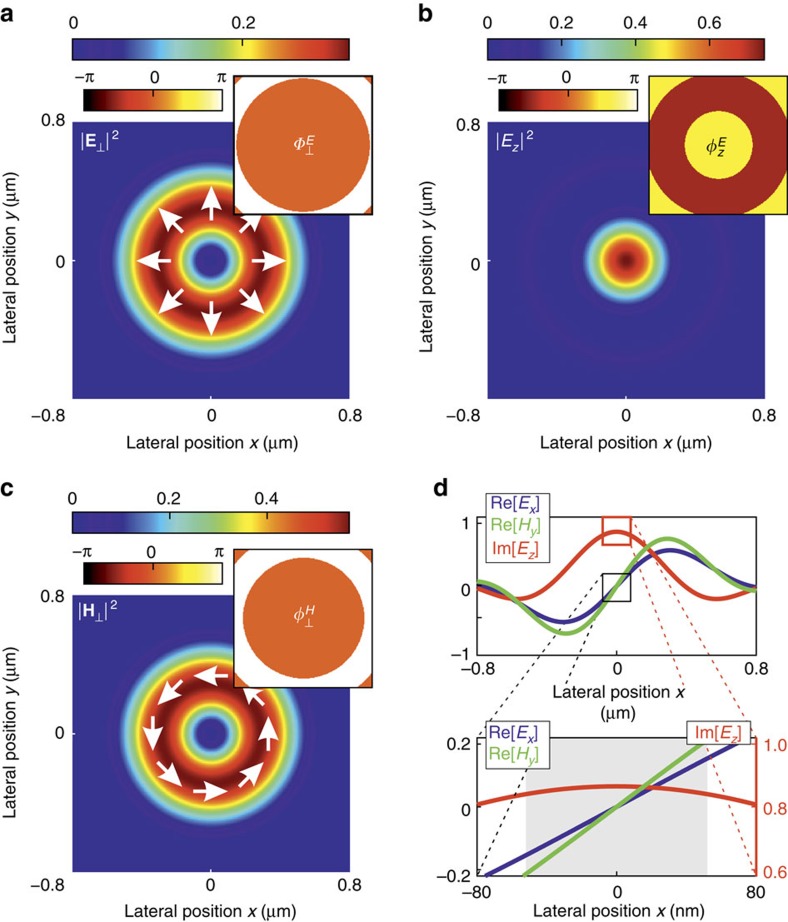
Theoretical field intensity distributions and relative phases of a tightly focused radially polarized beam. The wavelength *λ*=652 nm and experimental parameters are taken into account (see Methods section). (**a**) The transverse (radial) electric field intensity 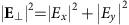, (**b**) the longitudinal electric field intensity 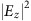 and (**c**) the transverse (azimuthal) magnetic field 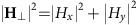 are all normalized to the maximum value of the total field intensity, 
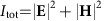
 (Gaussian units). The corresponding phase distributions 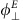, 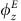 and 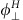 are plotted as insets. (**d**) Cross-sections of the focal fields along the *x* axis. Close to the centre (grey area in the lower image), the transverse field amplitudes Re[*E*_*x*_] and Re[*H*_*y*_] are linearly dependent on the position, while the longitudinal field Im[*E*_*z*_] is approximately constant.

It was shown that the simultaneous excitation of transverse electric and magnetic resonances of a high-refractive-index dielectric nanoparticle may yield enhanced or suppressed forward/backward scattering due to their interference^27^,^28^,^29^,^30^. However, by carefully structuring the excitation field three-dimensionally (3D) and thus also exciting longitudinal particle modes^9^, the scattering pattern can be tailored to achieve lateral directivity in the far field. For example, a tightly focused radially polarized beam features a promising 3D focal field with cylindrical symmetry^1^,^2^. Figure 1a–c shows its electric and magnetic field intensity distributions and the corresponding phases, calculated by vectorial diffraction theory^31^,^32^, while taking into account the experimental parameters. Apart from the transverse (in-plane) radially polarized electric field 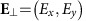, a strong longitudinal component *E*_*z*_ is formed, reaching its maximum amplitude on the optical axis. In contrast, the magnetic field 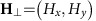 is purely transverse and azimuthally polarized. In close vicinity to the optical axis, *E*_*z*_ exhibits a phase delay of Δ*φ*_*z*_=±*π*/2 with respect to the transverse field components, and the electric and magnetic fields can be approximated by:


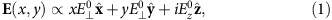



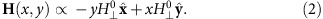


Here, 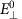, 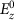 and 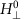 are real valued amplitudes of the transverse electric, longitudinal electric and transverse magnetic field components, respectively, and (*x*, *y*) are Cartesian coordinates in the focal plane. Without loss of generality, the point in time is chosen such that the transverse field components *E*_*x*_, *E*_*y*_, *H*_*x*_ and *H*_*y*_ are real, and the longitudinal component *E*_*z*_ is imaginary, owing to the aforementioned phase delay of *π*/2. For the chosen beam parameters (see Methods section), we estimate equations (1) and (2) to be valid within the region up to 50 nm away from the optical axis (see grey area in Fig. 1d). In this limited range, the transverse electric and magnetic fields are linearly dependent on the coordinates *x* and *y*, while *E*_*z*_ is assumed to be approximately constant. In order to adopt this linear position dependence of the transverse electromagnetic field for position sensing, a sub-wavelength antenna, capable of incorporating the local field in its far-field emission pattern, is required to localize the antenna unambiguously by its (directional) scattering pattern recorded in the far field. In the following, we discuss a silicon nanosphere (radius *r*=92 nm), whose spectrum in the visible range was experimentally investigated previously^9^, and we explain how its far-field emission pattern is governed by its position.

### Tailored directional scattering

**Figure 2:**
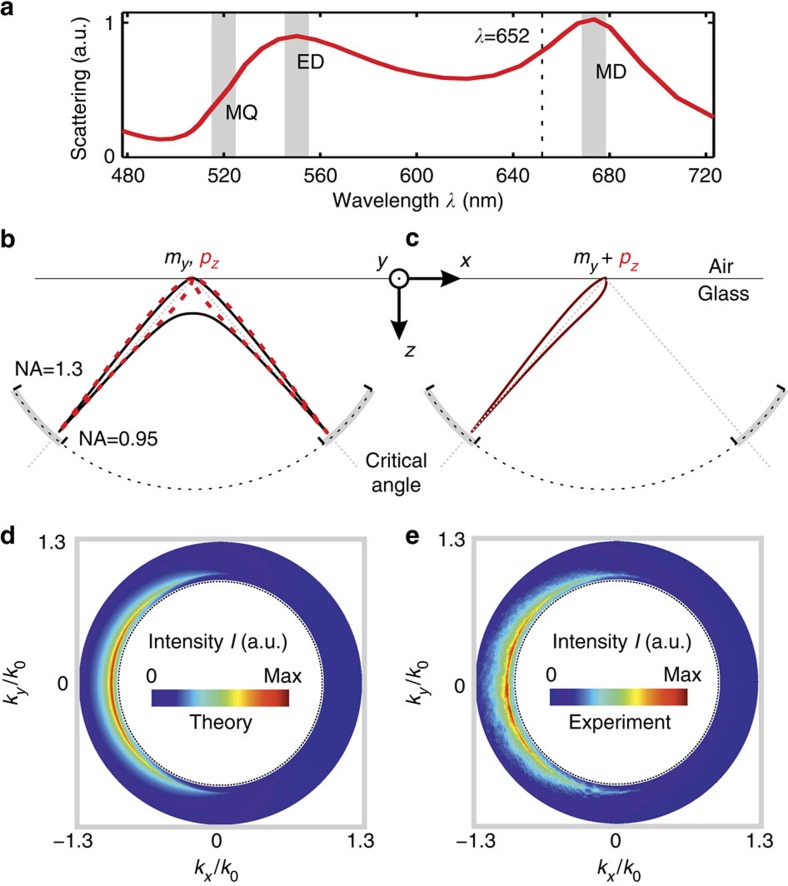
Scattering of a silicon nanoantenna sitting on a dielectric interface. (**a**) Simulated scattering cross-section (linearly polarized Gaussian beam used for excitation) of a silicon sphere with radius *r*=92 nm; only the forward scattering efficiency into the angular region within the NA∈[0.95, 1.3] is considered to match the experimental detection scheme (see grey arcs in **b**,**c**, and far-field patterns in **d**,**e**). In the visible range, the nanosphere supports magnetic dipole (*λ*_MD_≈670 nm), electric dipole (*λ*_ED_≈550 nm) and magnetic quadrupole (*λ*_MQ_≈520 nm) resonances. At the excitation wavelength of *λ*=652 nm, the magnetic and electric dipole moments are *π*/2 out of phase with respect to each other. (**b**) Emission of a longitudinal electric dipole *p*_*z*_ (see dashed red line) and a transverse magnetic dipole *m*_*y*_ (see black line) into the glass substrate. (**c**) In-phase far-field interference of *p*_*z*_ and *m*_*y*_ results in strong directivity. Comparison of (**d**) a calculated far-field pattern (interference of *p*_*z*_ and *m*_*y*_) and (**e**) a measured back-focal-plane image, retrieved at an antenna position on the *x* axis 140 nm away from the centre of the beam.

Figure 2a shows the scattering cross-section of the antenna sitting on a glass substrate, simulated using the finite-difference time-domain method (similar to ref. 9). Here, only the forward scattering efficiency into the angular region within the numerical aperture (NA)∈[0.95, 1.3] is considered to match the experimental detection scheme described below (see also Fig. 2b–e). In the visible spectral range, the silicon antenna supports the following three pronounced resonances:^9^ the magnetic dipole (*λ*_MD_≈670 nm), the electric dipole (*λ*_ED_≈540 nm) and the magnetic quadrupole (*λ*_MQ_≈515 nm). For wavelengths above 600 nm, the weak contribution of the magnetic quadrupole can be completely neglected^9^, and the antenna can be approximated by a point-like dipole (electric and magnetic). Assuming that the dipole moments are proportional to the respective local field vectors, **p**∝**E** and **m**∝**H**, we yield the position-dependent dipole moments 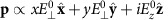 and 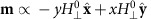. The aim of our experimental concept is to achieve highly position-sensitive far-field directivity caused by the interference of the, in first approximation, constant *z*-oriented electric dipole *p*_*z*_ and the position-dependent transverse components of the magnetic dipoles *m*_*x*_ and *m*_*y*_. The influence of the transverse electric dipole components *p*_*x*_ and *p*_*y*_ will be proven to be negligible later on.

In Fig. 2b, the far-field intensities of a *z*-oriented electric dipole (see dashed red line) and a *y*-oriented magnetic dipole (see black line) emitted into the glass substrate are depicted. Here, we consider the electric and magnetic dipole moments to exhibit the same strength. If the dipole moments are in phase, the interference of both far fields yields a remarkably strong lateral directivity (Fig. 2c). Figure 2d shows the corresponding calculated *k*-spectrum in the experimentally accessible region within NA∈[0.95, 1.3]. At this point, the relative phase between the longitudinal and the transverse field components (Δ*φ*_*z*_=±*π*/2, see insets in Fig. 1) of the excitation beam needs to be considered. If the electric and magnetic dipoles oscillate *π*/2 out of phase, no directivity would be observed in the far field because their symmetric far-field intensity distributions add up. Hence, an additional phase of *π*/2 is required to compensate for Δ*φ*_*z*_. Since the relative phase between a dipole moment and its respective excitation field (Δ*φ*_MD_ for the magnetic, Δ*φ*_ED_ for the electric field) depends on the wavelength, we can compensate for Δ*φ*_*z*_ by carefully choosing the wavelength of the incoming light with respect to the spectral positions of the electric and magnetic dipole resonances. From simulation, we retrieve the relative phase between the electric and magnetic dipole moment to be Δ*φ*=Δ*φ*_MD_−Δ*φ*_ED_=*π*/2 for a wavelength of *λ*=652 nm (Supplementary Fig. 1 and Supplementary Note 1). Using this wavelength for excitation, we expect to achieve strongly directional scattering at antenna positions where the longitudinal electric and transverse magnetic fields overlap. For experimental verification, a measured far-field image is plotted in Fig. 2e. The image was retrieved by placing the antenna on the *x* axis ∼140 nm away from the centre of the beam in the focal plane, effectively obtaining longitudinal electric and transverse magnetic dipole moments of equal strength. The strong directivity proves that the compensation of the relative phase has been successful, and the very good overlap of 94% with the theoretical pattern suggests that, in first-order approximation, the transverse electric dipole moments, not taken into account here, can indeed be neglected (details can be found in Supplementary Fig. 2 and Supplementary Note 2).

In short, we optimized the polarization distribution and the wavelength of our excitation beam to achieve strongly directional emission depending on the position of a single silicon nanoantenna relative to the beam's optical axis. The underlying principle causing the directivity is the simultaneous and in-phase excitation of a longitudinal electric and a transverse magnetic dipole moment.

### Experimental implementation and calibration

**Figure 3:**
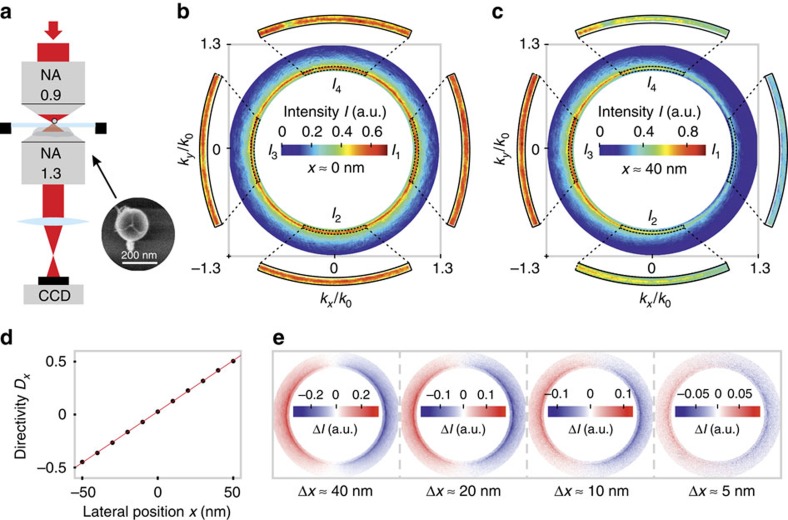
Set-up and experimental results. (**a**) A radially polarized collimated beam is tightly focused by a microscope objective (NA=0.9) onto a sub-wavelength silicon antenna sitting on a glass substrate (see inset). The light emitted into the angular regime with NA∈[0.95, 1.3] is collected by an oil-immersion type microscope objective (NA=1.3). The back-focal plane is imaged onto a CCD camera. (**b**) Acquired far-field intensity distribution *I*(*k*_*x*_, *k*_*y*_) for the antenna placed on the optical axis and (**c**) for the antenna displaced laterally by *x*≈40 nm (off-axis). The dashed black lines and the magnified insets indicate four regions with averaged intensities *I*_1_, *I*_2_, *I*_3_ and *I*_4_. (**d**) Directivity parameter *D*_*x*_ versus the antenna's position along the *x* axis; the slope of the curve (0.01 nm^−1^) defines the sensitivity of the measurement to the antenna displacement. (**e**) Far-field intensity difference images for four antenna positions along the *x* axis (left to right: Δ*x*≈40, 20, 10 and 5 nm away from the optical axis). The left image corresponds to the intensity difference Δ*I*(*k*_*x*_, *k*_*y*_) between **b**,**c**.

A sketch of the experimental set-up is depicted in Fig. 3a (for more details see ref. 33). The collimated incoming radially polarized beam (*λ*=652 nm) was tightly focused by a microscope objective with an NA of 0.9 onto the silicon nanosphere sitting on a glass substrate (see electron micrograph in Fig. 3a), which was positioned precisely within the focal plane by a 3D piezo-stage. A second microscope objective (oil-immersion type, NA=1.3) below the substrate collected both the transmitted beam and the forward scattered light. Imaging the back-focal plane of the second microscope objective onto a CCD camera enabled acquisition of the intensity distribution emitted into the far field and grants access to the angular spectrum of the scattered field (see examples in Figs 2e and 3b,c). Similar to ref. 10, only the region of NA∈[0.95, 1.3] was considered, where the scattered light can be detected without interfering with the transmitted beam.

To retrieve the position of the antenna from the back-focal plane images, we averaged the measured intensity over four small regions in *k*-space (black dotted lines in Fig. 3b,c)^34^, resulting in four averaged intensity values *I*_1_, *I*_2_, *I*_3_ and *I*_4_. The size and position of these four regions was chosen to include only the strongest change of the far-field intensity for an antenna shift along the *x* or *y* axis. The normalized intensity differences 
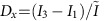
 and 
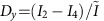
, with 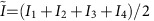, represent directivity parameters, which are linear functions of the antenna position (Supplementary Note 3).

In order to compensate for experimental imperfections such as beam aberrations or deviations from the ideal antenna shape, the measurement approach requires initial calibration, for which we placed the antenna centrally in the focus. At this position, the far-field distribution of the scattered light is expected to be cylindrically symmetric, since only a longitudinal electric dipole moment can be excited (Fig. 3b)^9^,^10^. From this reference point, the antenna was scanned across the focal plane (100 × 100 nm), with a step-size of 10 nm. For each position, an image of the back-focal plane was acquired and the corresponding values of *D*_*x*_ and *D*_*y*_ were determined. The whole procedure was repeated 40 times and the measured directivity parameters were averaged in order to decrease the influence of the instability of our set-up (position uncertainty of ±5 nm). Thereupon, linear equations were fitted to the averaged directivity parameters *D*_*x*_(*x*, *y*) and *D*_*y*_(*x*, *y*) (see equation (8) in the Methods section), which allow for retrieving the antenna position from individual back-focal plane images. As an example, Fig. 3d shows the averaged directivity parameter *D*_*x*_ plotted against the *x* coordinate and the corresponding linear fit.

### Lateral resolution

In order to demonstrate the accuracy in the measurement of the antenna position, which can be achieved with a single camera shot, far-field images for different antenna positions are analysed. To this end, we normalize the intensity maps recorded in each back-focal plane to the intensity 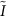 and calculate the difference to a reference image, which corresponds to the antenna sitting on the optical axis (Fig. 3b). For the demonstration of this inherently 2D localization technique, we show results for *x* displacements only. In Fig. 3e, we depict four post-selected difference images for the antenna being placed on the *x* axis, for which our calibration measurement indicated relative positions of Δ*x*≈40, 20, 10 and 5 nm (Δ*y*≈0 nm). For a relatively large displacement of Δ*x*≈40 nm, the difference image corresponding to the difference between Fig. 3b,c yields a very good signal-to-noise ratio. Even for a small displacement of only Δ*x*≈5 nm, the difference image reveals predominately negative values on the left side (*k*_*x*_<0) and positive values on the right side (*k*_*x*_>0). However, the signal-to-noise ratio decreases with shorter distances Δ*x*. The theoretical limit of our resolution is determined by the derivatives (slopes) of *D*_*x*_(*x*, *y*) and *D*_*y*_(*x*, *y*), the intensity noise of an individual camera pixel, and the actual number of pixels in each integration region. Our calculations yield that a position uncertainty below 2 Å could be achieved. More details and an actual experimental example can be found in Supplementary Fig. 3 and Supplementary Note 4. However, a direct proof of this accuracy would require a highly stabilized set-up including a piezo-stage with Ångström precision.

## Discussion

In summary, we experimentally demonstrated that the simultaneous and phase-adapted excitation of longitudinal electric and transverse magnetic dipole modes of a high-refractive-index nanosphere yields extraordinarily strong directionality. Especially the spectral tuning of the relative phase between both dipole modes in combination with the appropriate choice of a 3D focal field pattern enabled highly position-sensitive transverse scattering directionality. We utilized the approach as a novel technique for single-shot lateral position sensing, achieving localization accuracies down to a few Ångström, which is comparable to other state-of-the-art localization methods presented in literature^12^,^13^,^18^. Our technique could be applied for the stabilization of samples, for instance, in super-resolution microscopy. Furthermore, since the directionality is also present in the super-critical regime (NA>1), evanescent coupling to waveguide modes will allow for on-chip detection of the directional scattering and, hence, of the lateral position of the sample. Finally, future studies might demonstrate that antenna design and size, as well as the excitation field can be optimized to achieve an even stronger dependence of the directionality on the particle position, which would allow for sub-Ångström localization accuracies.

## Methods

### Experimental set-up

A tunable light source (NKT Photonics SuperK Extreme & SpectraK Dual) emits a linearly polarized Gaussian beam at a wavelength of 652 nm, which is converted into a radially polarized beam by a liquid-crystal polarization converter (q-plate)^35^,^36^. The beam with radius *w*_0_=1.26 mm is then guided into a microscope objective with NA=0.9 and an entrance aperture radius of 1.8 mm (Leica HCX PL FLUOTAR 100 × /0.90 POL 0/D). A single spherical silicon nanoparticle with radius *r*=92 nm on a glass substrate is scanned through the focal plane by a high-precision 3D piezo-stage (PI P-527), and the transmitted light is collected with an oil-immersion objective with NA=1.3 (Leica HCX PL FLUOTAR 100 × /1.30 OIL). The angular intensity distribution of the transmitted light is detected by imaging the back-focal plane of the oil-immersion objective onto a CCD camera (The Imaging Source DMK 23U618). The four solid angles corresponding to *I*_1_, *I*_2_, *I*_3_ and *I*_4_ (see dashed lines in Fig. 3b,c) are defined by an azimuthal angular range of ΔΦ=45° and by NA∈[0.98, 1.02].

### Calculation of the far-field distribution

We make use of the cylindrical symmetry of the beam and, without loss of generality, only consider antenna positions along the *x* axis. Therefore, only the longitudinal electric (*p*_*z*_) and transverse magnetic (*m*_*y*_) dipole moments need to be considered. The transverse electric (s-polarized) and transverse magnetic (p-polarized) far-field distributions 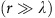 emitted into the dielectric substrate (refractive index *n*=1.5) are expressed in ref. 32.


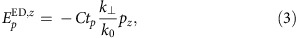



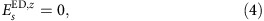



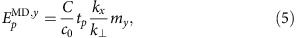



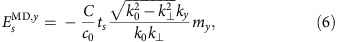


with


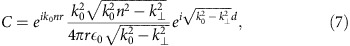


the Fresnel coefficients for transmission *t*_*p*_ and *t*_*s*_, the wavenumber in vacuum *k*_0_=2*π*/*λ*, the transverse component of the *k*-vector 
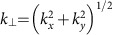
 and the vacuum speed of light *c*_0_. Comparison between theoretical and experimental far-field patterns enables estimating the distance between the effective point-like dipole and the interface, *d*=70 nm. The emission patterns in Fig. 2b,c are calculated using equations (3)–(6), 16, 17, 18, whereby we considered a similar strength for both dipole moments *p*_*z*_=*m*_*y*_/*c*_0_ to achieve maximum directivity. Taking into account the aplanatic microscope objective, an additional energy conservation factor proportional to 
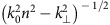
 is introduced for Fig. 2d (ref. 32).

### Calibration measurement

The directivity parameters *D*_*x*_ and *D*_*y*_ are linear functions of the lateral antenna position *x* and *y*, respectively. Thus, we fit a system of two linear equations to the averaged calibration measurement data, resulting in


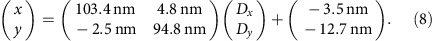


Ideally, the matrix has non-zero values on its diagonal only. The small off-diagonal elements indicate a minor rotation of the coordinate system and, in addition, not entirely orthogonal directivity parameters *D*_*x*_ and *D*_*y*_. The rotation of the coordinates might stem from a misalignment of our camera with respect to the coordinate frame of the piezo-stage, while the non-orthogonal basis can be related to aberrations of the beam and asymmetries of the antenna (see electron micrograph in Fig. 3a). The derivatives (slopes) of *D*_*x*_ and *D*_*y*_ define the sensitivity of the directivity to a displacement of the antenna. At the rim of the region of linearity, 50 nm away from the centre, we already achieve a directivity *D*_*x*_=48% (*D*_*y*_=52%) if the antenna is shifted in *x* direction (*y* direction).

### Post-selection of difference images

The instability of our experimental set-up causes an uncertainty of ±5 nm regarding the position of the particle relative to the beam. For this reason, we took 40 individual images for each position set by the piezo-stage (Δ*x*≈40, 20, 10 and 5 nm), and then post-selected the far-field images of which the directivity parameters *D*_*x*_ and *D*_*y*_ best-represented the position set by the piezo-stage according to the calibration measurement (equation (8)). Finally, we calculated the difference images depicted in Fig. 3e.

## Additional information

**How to cite this article:** Neugebauer, M. *et al*. Polarization-controlled directional scattering for nanoscopic position sensing. *Nat. Commun.* 7:11286 doi: 10.1038/ncomms11286 (2016).

## Supplementary Material

Supplementary Information Supplementary Figures 1-3, Supplementary Notes 1-4 and Supplementary References.

## References

[b1] Quabis S., Dorn R., Eberler M., Glöckl O. & Leuchs G. Focusing light to a tighter spot. Opt. Commun. 179, 1–7 (2000).

[b2] Youngworth K. & Brown T. Focusing of high numerical aperture cylindrical-vector beams. Opt. Express 7, 77–87 (2000). 19404372 10.1364/oe.7.000077

[b3] Wang H., Shi L., Lukyanchuk B., Sheppard C. & Chong C. T. Creation of a needle of longitudinally polarized light in vacuum using binary optics. Nat. Photon. 2, 501–505 (2008).

[b4] Chen R., Agarwal K., Sheppard C. J. R. & Chen X. Imaging using cylindrical vector beams in a high-numerical-aperture microscopy system. Opt. Lett. 38, 3111–3114 (2013). 24104662 10.1364/OL.38.003111

[b5] Rittweger E., Han K. Y., Irvine S. E., Eggeling C. & Hell S. W. STED microscopy reveals crystal colour centres with nanometric resolution. Nat. Photon. 3, 144–147 (2009).

[b6] Hao X., Kuang C., Wang T. & Liu X. Effects of polarization on the de-excitation dark focal spot in STED microscopy. J. Opt. 12, 115707–115714 (2010).

[b7] Yoshiki K., Hashtmoto M. & Araki T. Second-harmonic-generation microscopy using excitation beam with controlled polarization pattern to determine three-dimensional molecular orientation. Jpn J. Appl. Phys. 44, 32–35 (2005).

[b8] Sancho-Parramon J. & Bosch S. Dark modes and Fano resonances in plasmonic clusters excited by cylindrical vector beams. ACS Nano 6, 8415–8423 (2012). 22920735 10.1021/nn303243p

[b9] Woźniak P., Banzer P. & Leuchs G. Selective switching of individual multipole resonances in single dielectric nanoparticles. Laser Photon. Rev. 9, 231–240 (2015).

[b10] Neugebauer M., Bauer T., Banzer P. & Leuchs G. Polarization tailored light driven directional optical nanobeacon. Nano Lett. 14, 2546–2551 (2014). 24724814 10.1021/nl5003526

[b11] Gelles J., Schnapp B. J. & Sheetz M. P. Tracking kinesin-driven movements with nanometre-scale precision. Nature 331, 450–453 (1988). 3123999 10.1038/331450a0

[b12] Nugent-Glandorf L. & Perkins T. T. Measuring 0.1-nm motion in 1 ms in an optical microscope with differential back-focal-plane detection. Opt. Lett. 29, 2611–2613 (2004). 15552661 10.1364/ol.29.002611

[b13] Rohrbach A. & Stelzer E. H. K. Three-dimensional position detection of optically trapped dielectric particles. J. Appl. Phys. 91, 5474–5488 (2002).

[b14] Gittes F. & Schmidt C. F. Interference model for back-focal-plane displacement detection in optical tweezers. Opt. Lett. 23, 7–9 (1998). 18084394 10.1364/ol.23.000007

[b15] Dupont A. & Lamb D. C. Nanoscale three-dimensional single particle tracking. Nanoscale 3, 4532–4541 (2011). 21960183 10.1039/c1nr10989h

[b16] Weisenburger S. . Cryogenic colocalization microscopy for nanometer-distance measurements. Chemphyschem 15, 763–770 (2014). 24677759 10.1002/cphc.201301080

[b17] Roy S., Ushakova K., van den Berg Q., Pereira S. F. & Urbach H. P. Radially polarized light for detection and nanolocalization of dielectric particles on a planar substrate. Phys. Rev. Lett. 114, 103903–103907 (2015). 25815935 10.1103/PhysRevLett.114.103903

[b18] Bon P. . Three-dimensional nanometre localization of nanoparticles to enhance super-resolution microscopy. Nat. Commun. 6, 7764 (2015). 26212705 10.1038/ncomms8764 PMC4525210

[b19] Hell S. W. & Wichmann J. Breaking the diffraction resolution limit by stimulated emission: stimulated-emission-depletion fluorescence microscopy. Opt. Lett. 19, 780–782 (1994). 19844443 10.1364/ol.19.000780

[b20] Klar T. A. & Hell S. W. Subdiffraction resolution in far-field fluorescence microscopy. Opt. Lett. 24, 954–956 (1999). 18073907 10.1364/ol.24.000954

[b21] Hess S. T., Girirajan T. P. K. & Mason M. D. Ultra-high resoluion imaging by fluorescence photoactivation localization microscopy. Biophys. J. 91, 4258–4272 (2006). 16980368 10.1529/biophysj.106.091116 PMC1635685

[b22] Betzig E. . Imaging intracellular fluorescent proteins at nanometer resolution. Science 313, 1642–1645 (2006). 16902090 10.1126/science.1127344

[b23] Evlyukhin A. B. . Demonstration of magnetic dipole resonances of dielectric nanospheres in the visible region. Nano Lett. 12, 3749–3755 (2012). 22703443 10.1021/nl301594s

[b24] Shi L., Tuzer T. U., Fenollosa R. & Meseguer F. A new dielectric metamaterial building block with a strong magnetic response in the sub-1.5-micrometer region: Silicon colloid nanocavities. Adv. Mater. 24, 5934–5938 (2012). 22927242 10.1002/adma.201201987

[b25] Bakker R. M. . Magnetic and electric hotspots with silicon nanodimers. Nano Lett. 15, 2137–2142 (2015). 25686205 10.1021/acs.nanolett.5b00128

[b26] Albella P., Shibanuma T. & Maier S. A. Switchable directional scattering of electromagnetic radiation with subwavelength asymmetric silicon dimers. Sci. Rep. 5, 18322 (2015). 26656864 10.1038/srep18322 PMC4674758

[b27] Kerker M., Wang D.-S. & Giles C. L. Electromagnetic scattering by magnetic spheres. J. Opt. Soc. Am. 73, 765–767 (1983).

[b28] Garca-Cámara B., de la Osa R. A., Saiz J. M., González F. & Moreno F. Directionality in scattering by nanoparticles: Kerker's null-scattering conditions revisited. Opt. Lett. 36, 728–730 (2011). 21368963 10.1364/OL.36.000728

[b29] Fu Y. H., Kuznetsov A. I., Miroshnichenko A. E., Yu Y. F. & Luk'yanchuk B. Directional visible light scattering by silicon nanoparticles. Nat. Commun. 4, 1527 (2013). 23443555 10.1038/ncomms2538

[b30] Alaee R. . All-dielectric reciprocal bianisotropic nanoparticles. Phys. Rev. B 92, 245130–245135 (2015).

[b31] Richards B. & Wolf E. Electromagnetic diffraction in optical systems. II. Structure of the image field in an aplanatic system. Proc. R. Soc. Lond. A 253, 358–379 (1959).

[b32] Novotny L. & Hecht B. Principles of Nano-Optics Cambridge University Press (2006).

[b33] Banzer P., Peschel U., Quabis S. & Leuchs G. On the experimental investigation of the electric and magnetic response of a single nano-structure. Opt. Express 18, 10905–10923 (2010). 20588946 10.1364/OE.18.010905

[b34] Neugebauer M., Bauer T., Aiello A. & Banzer P. Measuring the transverse spin density of light. Phys. Rev. Lett. 114, 063901–063901 (2015). 25723220 10.1103/PhysRevLett.114.063901

[b35] Marrucci L., Manzo C. & Paparo D. Optical spin-to-orbital angular momentum conversion in inhomogeneous anisotropic media. Phys. Rev. Lett. 96, 163905–163908 (2006). 16712234 10.1103/PhysRevLett.96.163905

[b36] Slussarenko S. . Tunable liquid crystal q-plates with arbitrary topological charge. Opt. Express 19, 4085–4090 (2011). 21369237 10.1364/OE.19.004085

