# Supplementary material for: Polarization-controlled directional scattering for nanoscopic position sensing
Source: Nat Commun. 2016 Apr 20;7:11286. doi: 10.1038/ncomms11286 (PMC4842979; doi:10.1038/ncomms11286)
Supplement: Supplementary Information — Supplementary Figures 1-3, Supplementary Notes 1-4 and Supplementary References. [file ncomms11286-s1.pdf]

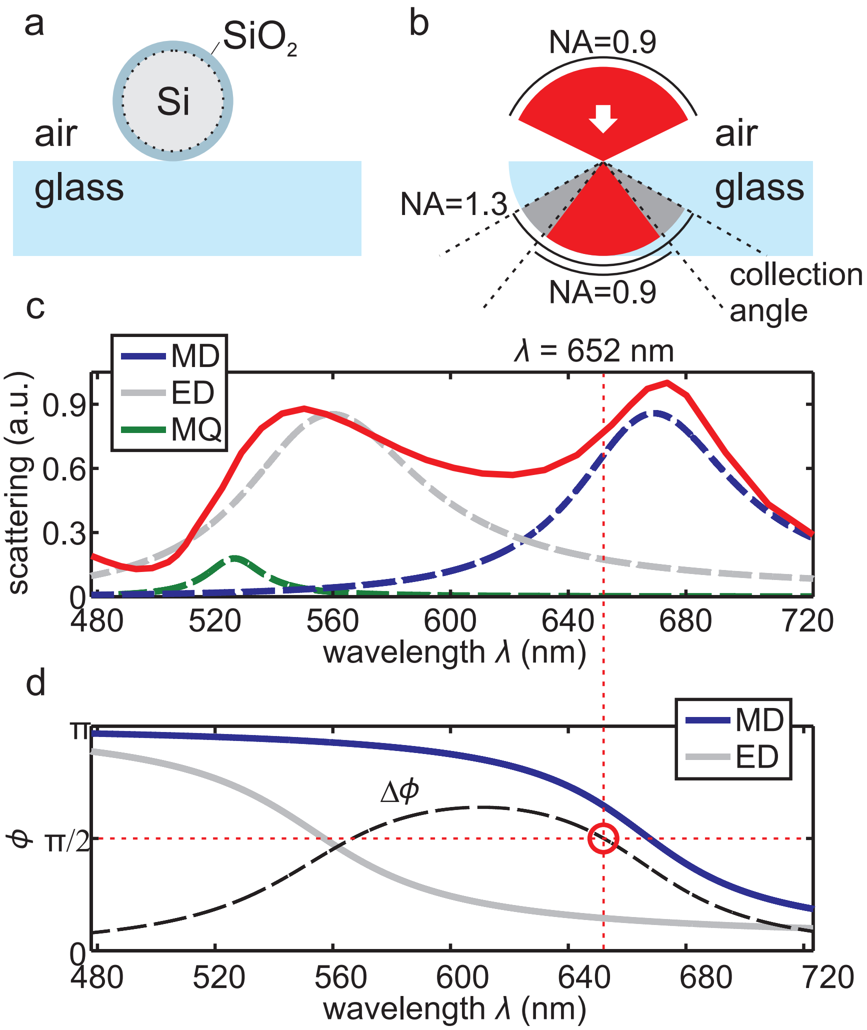

Supplementary Figure 1: Phase retrieval from simulation. **a** Sketch of the silicon (Si) antenna model with silicon dioxide (SiO_2) shell used for simulation. **b** Numerical apertures of the microscope objectives used for focusing the incoming beam (NA = 0.9) and collecting the scattered and transmitted light (NA = 1.3). The actual collection angle is set to NA $\in [0.95, 1.3]$. **c** Scattering cross-section as depicted in Fig. 2 of the main manuscript (see solid red line). A sum of three Lorenzians representing the magnetic dipole MD (dashed blue line), electric dipole ED (dashed gray line) and the magnetic quadrupole MQ (dashed green line) is fitted to the simulated spectrum. **d** Relative phases between the excitation field and the corresponding magnetic (ϕ_{MD}, solid blue line) and electric (ϕ_{ED}, solid gray line) dipole moments. At the chosen excitation wavelength of $\lambda = 652$ nm, the phase difference $\Delta\phi = \phi_{MD} - \phi_{ED}$ (dashed black line) is $\pi/2$.

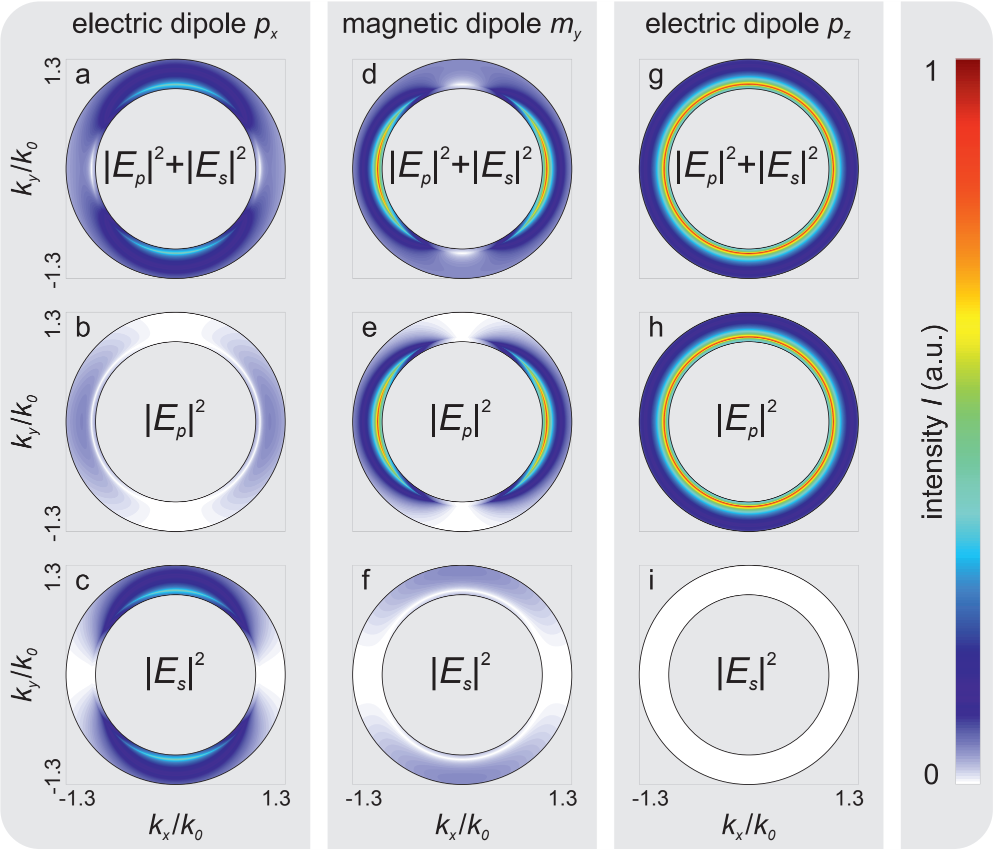

Supplementary Figure 2: Negligibility of the transverse electric dipole. The far-field intensity patterns of the dipole moments p_x, m_y and p_z emitted into the glass substrate are calculated (distance between the effective dipole and the glass interface $d = 70$ nm). Similar to the experiment, only the angular region within NA $\in [0.95, 1.3]$ is considered. **a**, **d** and **g** illustrate the total far-field intensities $I = |E_p|^2 + |E_s|^2$, with **b-c**, **e-f** and **h-i** depicting the individual far-field polarization components $|E_p|^2$ and $|E_s|^2$, respectively. All intensity distributions are normalized to the maximum of **g**.

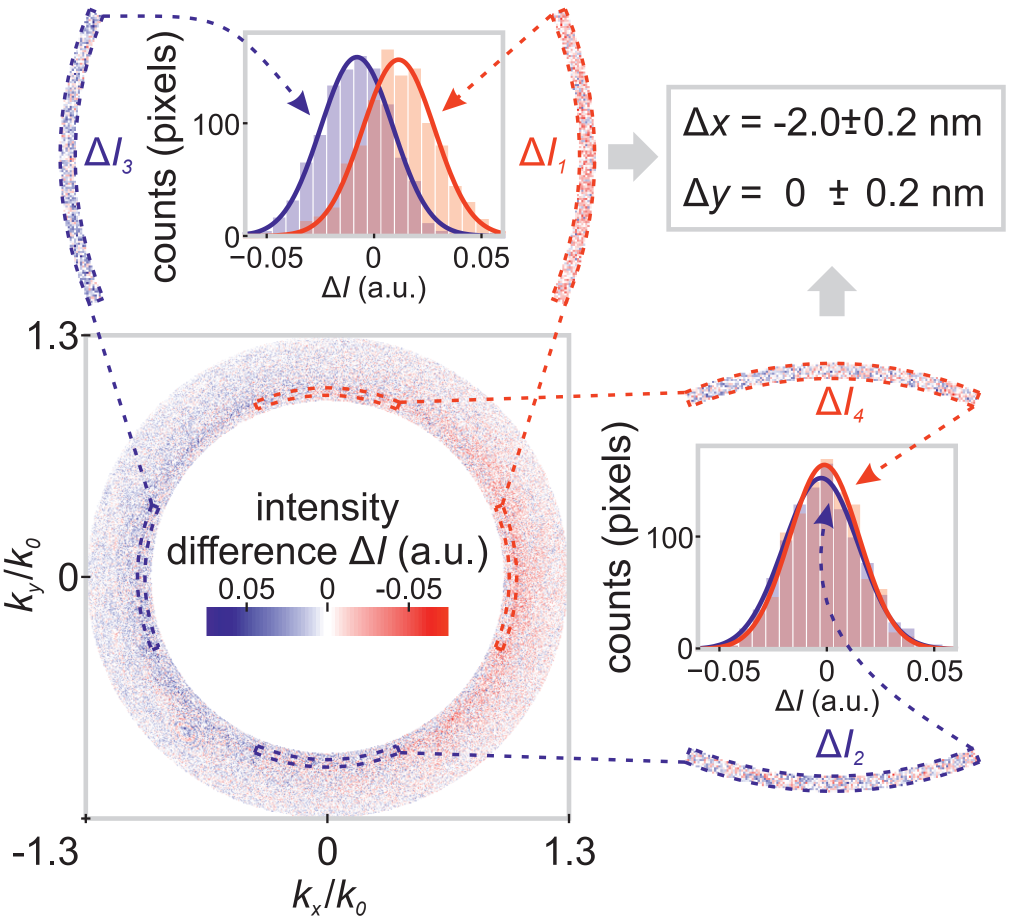

Supplementary Figure 3: Estimation of the resolution. The difference image of two back-focal plane images with similar intensity distributions ($\Delta x \approx -2$ nm, $\Delta y \approx 0$ nm) is depicted. The statistics of the camera noise in each region (histograms shown as insets) can be used to determine the resolution of our experiment.

SUPPLEMENTARY NOTE 1

Phase retrieval from simulation

Figure 2a of the main manuscript and Supplementary Fig. 1c show the scattering cross-section of the silicon (Si) antenna sitting on a glass substrate, simulated using the finite-difference time-domain method. Similar to refs. [1–3], our Si nanosphere with an outer diameter of 184 nm is modeled including a thin silicon dioxide (SiO_2) surface layer with an approximated thickness of 8 nm due to oxidation (see sketch in Supplementary Fig. 1a). The material properties of Si and SiO_2 are adopted from the database of Palik [4]. In general, Si has a high refractive index and a small extinction coefficient in the visible regime (e.g. $n_{Si} = 3.85 - 0.02i$ at $\lambda = 652$nm). In order to adapt the simulation to the experiment, we consider a tightly focused linearly polarized Gaussian beam as a source (maximum NA = 0.9), and collect only the light scattered in the forward direction into the far-field with a polar collection angle set to NA $\in [0.95, 1.3]$ (see sketch in Supplementary Fig. 1b). In the investigated spectral regime, a Si nanosphere of the chosen size placed on a substrate supports three pronounced resonances, the magnetic dipole ($\lambda_{\mathrm{MD}} \approx 660$ nm), the electric dipole ($\lambda_{\mathrm{ED}} \approx 540$ nm) and the magnetic quadrupole ($\lambda_{\mathrm{MQ}} \approx 515$ nm) [3]. For this reason, the scattering spectrum (see red line in Supplementary Fig. 1c) can be described, in first approximation, by the sum of three individual Lorentzian curves, fitted to the simulation. The weak contribution of the magnetic quadrupole (dashed green line) can be neglected for wavelengths above 600 nm. Therefore, only electric (dashed gray line) and magnetic (dashed blue line) dipole resonances need to be considered for the chosen excitation wavelength of 652 nm. Now, we consider the relative phases of the magnetic and electric dipole moments with respect to their corresponding excitation fields. Following the Lorentz oscillator model [5], the relative phases of the electric (ϕ_{ED}) and magnetic (ϕ_{MD}) dipole moments depend on the excitation wavelength. Supplementary Fig. 1d shows ϕ_{ED} (gray line), ϕ_{MD} (blue line), and the phase difference $\Delta\phi = \phi_{MD} - \phi_{ED}$ (dashed black line). We find two wavelengths with $\Delta\phi = \pi/2$. However, we choose the wavelength 652 nm, which is close to λ_{MD} and guarantees a sufficient overlap between both types of dipoles (see dashed vertical and horizontal red lines). As mentioned above, the magnetic quadrupole can be neglected for the chosen wavelength. Another advantage of this choice of wavelength close to the

magnetic resonance is the much higher efficiency, with which the magnetic dipole mode can be excited in comparison to its electric counterpart. This leads to comparable scattering signal strengths from both induced magnetic and electric dipole moments (even though the electric field is much stronger than the magnetic field close to the optical axis) and, therefore, stronger asymmetry upon interference. Consequently, an enhanced position dependence of the directionality is realized.

SUPPLEMENTARY NOTE 2

Negligibility of the transverse electric dipole

As mentioned in the main manuscript, we expect the electric and magnetic dipole moments to be proportional to the respective local field vectors, $\mathbf{p} \propto \mathbf{E}$ and $\mathbf{m} \propto \mathbf{H}$. This includes the transverse electric dipole moments $p_x \propto E_x$ and $p_y \propto E_y$. However, for several reasons we can neglect the influence of the transverse electric dipole moments in first approximation.

First of all, even at the rim of the region of linearity, roughly 50 nm away from the optical axis, the longitudinal electric field is still stronger than the transverse ones by a factor of 4 (see Fig. 1d in the manuscript). Second, with the given excitation wavelength close to the magnetic dipole resonance of the antenna, we expect to excite the transverse magnetic dipole moment with a higher efficiency than the transverse electric dipole moment (see Supplementary Fig. 1). A third reason is based on the actual far-field emission patterns of the individual dipole moments plotted in Supplementary Figs. 2a-i. Similar to ref. [6], the lateral directivity is linked to the transverse magnetic far-field component E_p. However, only p_z is emitting exclusively as E_p (see Eqs. 3 and 4 of the main manuscript and Supplementary Figs. 2h and i). The influence of the individual dipole moments on the directivity is therefore governed by the amount of light emitted into E_p. For each dipole we calculate the power of the transverse magnetic field component by integrating over the distributions plotted in Supplementary Figs. 2b, e and h. The ratios between these power values for p_x, m_y and p_z equals to $1 : 6.4 : 14.5$. We conclude that the influence of transverse electric dipole moments can indeed be neglected in first approximation. In particular, its influence on the directivity parameters is very small.

SUPPLEMENTARY NOTE 3

Law of proportionality for the directivity parameters

The directivity parameters D_x and D_y correspond to differences of averaged intensity values recorded in the far-field. In order to derive equations describing the position dependence of D_x and D_y, we first calculate the far-field intensity patterns $I(k_x, k_y)$ depending on the longitudinal electric dipole moment ($p_z \in \mathbb{R}$) and the transverse magnetic dipole moments ($m_x, m_y \in \mathbb{R}$). As mentioned in the main manuscript, we neglect any influence of the transverse electric dipole moments as well as the magnetic quadrupole (see Supplementary Notes 1 and 2). Because of the cylindrical symmetry of the excitation beam and without loss of generality, we only consider antenna positions along the x-axis ($m_x = 0$). The far-field intensity distribution of the light emitted into the glass substrate can be written as $I(k_x, k_y) = |E_p|^2 + |E_s|^2$, with

$$E_p = E_p^{ED,z} + E_p^{MD,y}, \tag{S1}$$

$$E_s = E_s^{ED,z} + E_s^{MD,y}. \tag{S2}$$

From Eqs. 3-6 in the Methods section of the main manuscript it follows

$$I(k_x, k_y) = |Ct_p|^2 \left[\left(\frac{k_\perp}{k_0} p_z \right)^2 + \left(\frac{k_x}{c_0 k_\perp} m_y \right)^2 - 2 \frac{k_x}{c_0 k_0} p_z m_y \right] + |Ct_s|^2 \left| \frac{\sqrt{k_0^2 - k_\perp^2}\, k_y}{c_0 k_0 k_\perp} m_y \right|^2 . \tag{S3}$$

Experimentally, we only consider the far-field intensity close to the critical angle I_c, which implies $k_\perp \approx k_0$ and simplifies Eq. S3 to

$$I_c(k_x, k_y) = |Ct_p|^2 \left[p_z^2 + \left(\frac{k_x}{c_0 k_0} m_y \right)^2 - 2 \frac{k_x}{c_0 k_0} p_z m_y \right] . \tag{S4}$$

The difference between two intensity values with respect to the y-axis yields

$$I_c(-k_x, k_y) - I_c(k_x, k_y) = 4 |Ct_p|^2 \frac{k_x}{c_0 k_0} p_z m_y. \tag{S5}$$

Since only the transverse magnetic dipole depends on the position as $m_y \propto x H_\perp^0$ (see manuscript), it directly follows that $D_x \propto I_c(-k_x, k_y) - I_c(k_x, k_y) \propto x$. The result can also be extended to the two-dimensional case with $D_y \propto I_c(k_x, -k_y) - I_c(k_x, k_y) \propto y$.

SUPPLEMENTARY NOTE 4

Estimation of the resolution

In order to estimate the resolution of our position sensing experiment, we compare two post-selected nearly identical far-field images (see difference image in Supplementary Fig. 3) and calculate the average intensity differences for all four regions, $\Delta I_1 = 11 \cdot 10^{-3}$, $\Delta I_2 = -10^{-3}$, $\Delta I_3 = -8 \cdot 10^{-3}$, $\Delta I_4 = -10^{-3}$. The corresponding standard deviations ($\sigma_i \approx 25 \cdot 10^{-3}$ for $i \in [1, 4]$, see histograms plotted as insets in Supplementary Fig. 3) and the number of pixels in each region (1050 pixels), yield an uncertainty of the mean intensities of $\pm 10^{-3}$ for each intensity value. These results indicate that a relative shift of the antenna's position of $\Delta x = -2 \pm 0.2$ nm and $\Delta y = 0 \pm 0.2$ nm was measured with an uncertainty in the Ångström regime. Hence, the two almost identical back focal plane images used for this estimation correspond to two antenna positions, which were different by only 2 ± 0.2 nm.

---

[1] Evlyukhin, A. B., Novikov, S. M., Zywietz, U., Eriksen, R. L., Reinhardt, C., Bozhevolnyi, S. I. & Chichkov, B. N. Demonstration of magnetic dipole resonances of dielectric nanospheres in the visible region. *Nano Lett.* **12,** 3749–3755 (2012).

[2] Fu, Y. H., Kuznetsov, A. I., Miroshnichenko, A. E., Yu, Y. F. & Luk'yanchuk, B. Directional visible light scattering by silicon nanoparticles. *Nature Commun.* **4,** 1527 (2013).

[3] Woźniak, P., Banzer, P. & Leuchs, G. Selective switching of individual multipole resonances in single dielectric nanoparticles. *Laser Photon. Rev.* **9,** 231–240 (2015).

[4] Palik, E. D. *Handbook of Optical Constants of Solids.* Academic Press, San Diego (1995).

[5] Joe, Y. S., Satanin, A. M. & Kim, C. S. Classical analogy of Fano resonances. *Phys. Scripta* **74,** 259–266 (2006).

[6] Neugebauer, M., Bauer, T., Banzer, P. & Leuchs, G. Polarization tailored light driven directional optical nanobeacon. *Nano Lett.* **14,** 2546–2551 (2014).
